# Differentiating snail intermediate hosts of *Schistosoma* spp. using molecular approaches: fundamental to successful integrated control mechanism in Africa

**DOI:** 10.1186/s40249-018-0401-z

**Published:** 2018-03-26

**Authors:** Eniola Michael Abe, Wei Guan, Yun-Hai Guo, Kokouvi Kassegne, Zhi-Qiang Qin, Jing Xu, Jun-Hu Chen, Uwem Friday Ekpo, Shi-Zhu Li, Xiao-Nong Zhou

**Affiliations:** 1 National Institute of Parasitic Diseases, Chinese Center for Disease Control and Prevention; Key Laboratory of Parasite and Vector Biology, MOH ; National Center for International Research on Tropical Diseases, Ministry of Science and Technology; WHO Collaborating Centre for Tropical Diseases, Shanghai, 200020 China; 2 Department of Pure & Applied Zoology, Federal University of Agriculture Abeokuta, Abeokuta, Nigeria

**Keywords:** Schistosomiasis, snail host, *schistosoma* spp., genome database, Africa

## Abstract

**Background:**

Snail intermediate hosts play active roles in the transmission of snail-borne trematode infections in Africa. A good knowledge of snail-borne diseases epidemiology particularly snail intermediate host populations would provide the necessary impetus to complementing existing control strategy.

**Main body:**

This review highlights the importance of molecular approaches in differentiating snail hosts population structure and the need to provide adequate information on snail host populations by updating snail hosts genome database for Africa, in order to equip different stakeholders with adequate information on the ecology of snail intermediate hosts and their roles in the transmission of different diseases. Also, we identify the gaps and areas where there is need for urgent intervention to facilitate effective integrated control of schistosomiasis and other snail-borne trematode infections.

**Conclusions:**

Prioritizing snail studies, especially snail differentiation using molecular tools will boost disease surveillance and also enhance efficient schistosomaisis control programme in Africa.

**Electronic supplementary material:**

The online version of this article (10.1186/s40249-018-0401-z) contains supplementary material, which is available to authorized users.

## Multilingual abstracts

Please see Additional file 1 for translations of the abstract into the four official working languages of the United Nations.

## Background

Snails are invertebrate animals, belonging to the Phylum Mollusca. This group of organisms (except slug) possess a unique feature, known as “shell” which is a major characteristic of the group [1]. The snails inhabits a wide range of habitats because they are found not only in freshwater environment but also in other ecological niches [2].

Some snails are medically important because they transmit disease-causing trematodes in humans and other animals [3]. Most of the diseases caused by snail-borne trematodes are prevalent in the tropic and sub-tropic regions of the world, and the medical and economic burden of these diseases are often neglected which is why they are called neglected tropical diseases (NTDs). The distribution of the diseases caused by snail-borne trematodes especially schistosomiasis is focal. Hence, the parasites distribution is strongly dependent on the intermediate snail hosts distribution [4, 5].

Firstly, the continued transmission of snail-borne trematode infections in most endemic areas is facilitated by the presence and distribution [6, 7] of these important snail intermediate hosts that provide suitable environment for the development of trematode parasites [8].

Secondly, poor access to basic infrastructure by most inhabitants living in endemic settings [9] and the limitation of chemotherapy (the main control strategy in Africa) to effectively control the burden of schistosomiasis led to the call for the implementation of integrated control strategies through the incorporation of snail control to achieve the goal of schistosomiasis elimination [3]. More so, the high risk population largely depend on water bodies domiciled by the snail hosts for their daily and economic activities.

Several studies have been done to unravel the identity of the “supposed enemy” whose influence is of great public health importance and with pronounced burden amongst people living in marginalized settings of the tropic and sub-tropic regions [10–12].

Therefore, it is important to develop the platform that will monitor and identify snail distribution and infected snails, to help improve control efforts of the diseases caused by snail-borne trematodes. Also, a lot of achievements have been recorded in the identification of some snail hosts of medical and veterinary importance using both morphological and molecular approaches [13–16] and these have provided information that helped improve schistosomiasis control efforts.

There are continued efforts at improving the development of biomarkers that are effective in differentiating schistosome parasites and also provide insights into factors influencing host-parasites compatibilities on local scales [17, 18].

Despite all the efforts, it is obvious that more reliable genomic information is required for snail intermediate hosts populations to help improve control programmes [19, 20] particularly in the schistosomiasis endemic regions of Africa. It is imperative to develop tools that will detect and quantify genetic differences and changes in snail populations and also closely monitor the spread of these genetic variants that have the potentials to affect control strategies [21].

Great tasks lie ahead and more commitment is required to ensuring the elimination of schistosomiasis from endemic regions of Africa.

Though, snail hosts studies are crucial especially in Africa as we prioritize NTDs elimination but only few studies have established snail hosts differentiation on local scales [15, 22, 23].

Therefore, it is imperative to provide adequate information on snail host population structure and diversity both on national and continental scales using molecular approaches in order to strengthen control programmes in Africa. Such information is important for reliable decision making and efficient control implementation. This should be a pre-requisite for setting up effective control programmes that will be supported by active surveillance response system in endemic areas especially in sub-Saharan Africa where the disease burden is enormous but control efforts are limited due to poor funding and lack of political will.

As suggested by Rollinson et al. [24] that a global awareness be raised to provide adequate support for the elimination of schistosomiasis in endemic countries, it is believed that the support will be more effective by updating the genomic status of snail hosts of trematode parasites where available and also establish reliable comprehensive genome identification database where information is lacking across Africa.

This paper summarize the available information on the progress made in controlling schistosomiasis transmission through snail intermediate hosts studies using molecular approaches and also identify areas where actions are required to be taken for effective integrated control efforts to be achieved in Africa.

The predominant snail intermediate hosts implicated for transmitting schistosome parasites in Africa is shown in Table 1.

**Table 1:** Predominant snail intermediate hosts found in Africa and the schistosome parasites harboured by them

S/N	Snail intermediate hosts	Parasites transmitted
1	*Bulinus globosus*	*Schistosoma haematobium*
2	*Bulinus truncatus*	*Schistosoma haematobium*
3	*Bulinus africanus*	*Schistosoma haematobium*
4	*Bulinus senegalensis*	*Schistosoma haematobium*
5	*Bulinus forskalii*	Potential snail intermediate host
6	*Bulinus camerunensis*	*Schistosoma haematobium*
7	*Biomphalaria pfeifferi*	*Schistosoma mansoni*
8	*Biomphalaria sudanica*	*Schistosoma mansoni*
9	*Biomphalaria choanomphala*	*Schistosoma mansoni*
10	*Bulinus alexandrina*	*Schistosoma mansoni*

Source: [25] http://www.cdc.gov/parasites/schistosomiasis/biology.html

**Fig. 1:**
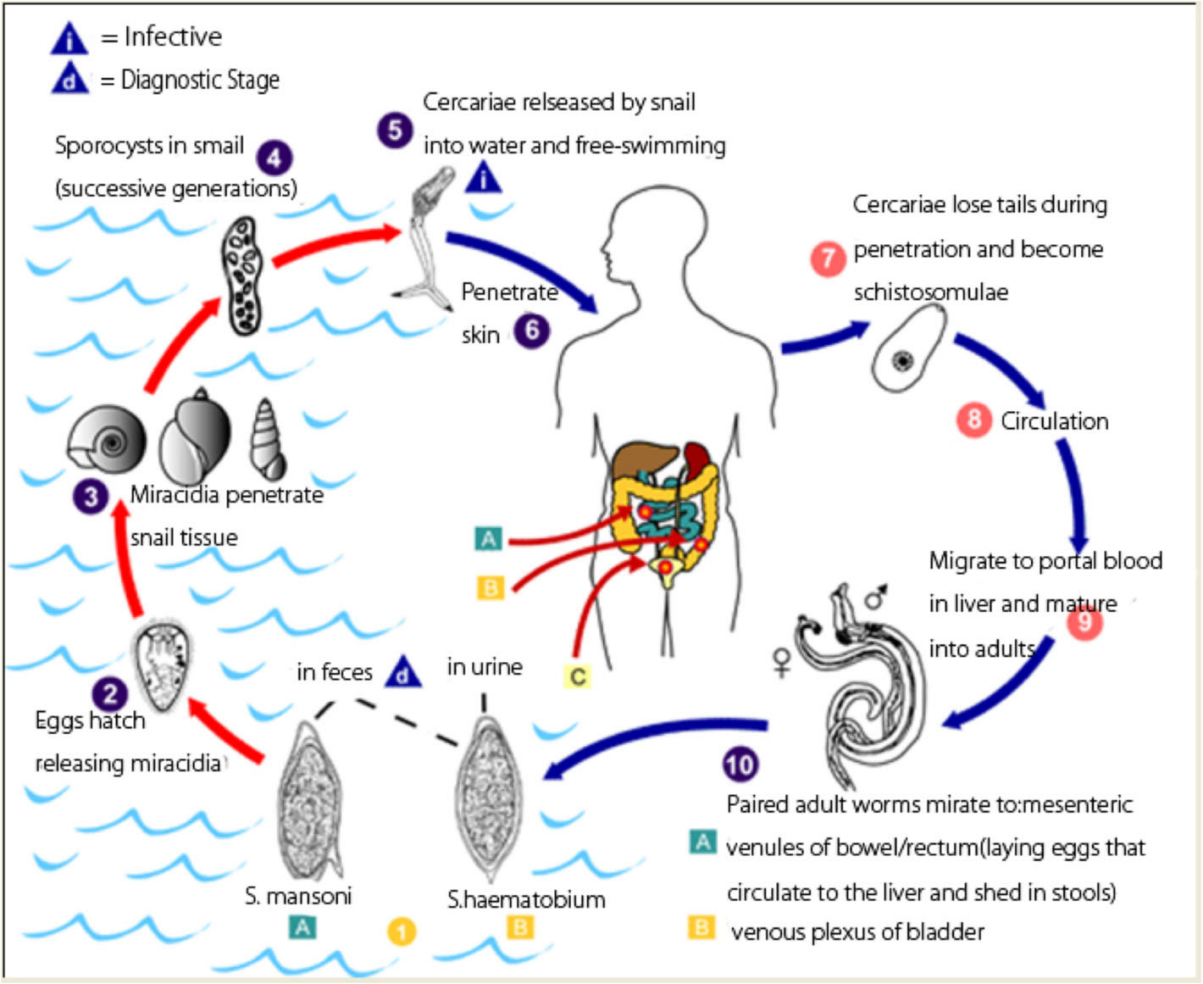
Typical life cycle of schistosome parasites [84]

The male and female adult schistosome worms dwell inside the blood stream of humans. *Schistosoma mansoni* and *S. haematobium* are responsible for intestinal schistosomiasis and urinary schistosomiasis respectively [25] (Fig. 1). *S. haematobium* is located in the venous plexus and it drains the infected person’s urinary bladder while *S. mansoni* is located in the mesenteric veins and it drains both the large and small instestines.

Schistosome eggs equipped with spines are deposited by the female schistosome parasites into the small venules of the portal and perivesical systems. The eggs migrate towards the bladder and ureter (*S. haematobium*) and the lumen of the intestine (*S. mansoni*) and are released into the environment with urine or feces. The accumulation of eggs deposited in the venules cause its blockage and this burst the veins and allows eggs and blood to access the urinary bladder and the intestine and this leads to the characteristic symptom of blood in urine and feces. When the eggs are released into the freshwater bodies, they hatch into miracidia and penetrate a suitable snail intermediate host of the genus *Bulinus* (with species such as *Bulinus truncatus, B. globosus, B. senegalensis, B. forskalii, B. camerunensis, B. africanus* and *B. tropicus*) or *Biomphalaria* (with species such as *Biomphalaria pfeifferi, Bi. Choanomphala, Bi. alexandrina, Bi. sudanica*), both serve as snail hosts of *S. haematobium* and *S. mansoni* respectively. The schistosome parasites develop and multiply into the infective cercariae within the snail hosts and are released into the water bodies by the snails. Humans become infected when they have contact with waterbodies that are infested with active cercariae [25].

**Fig. 2:**
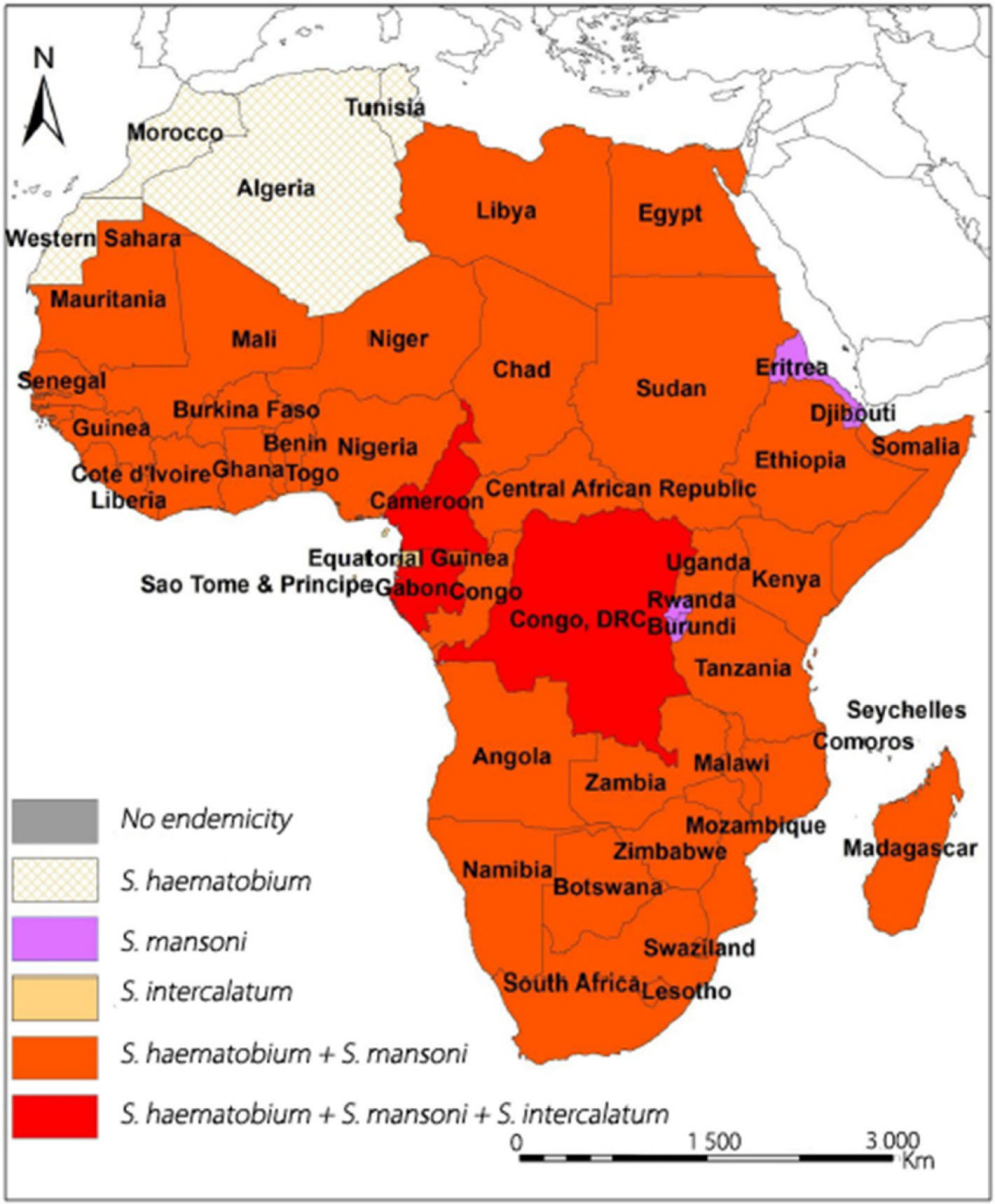
Distribution of schistosomiasis in Africa [26]

Figure 2 shows the distribution of schistosomiasis on the African continent [26].

Tables 2 and 3 shows the identified *Bulinus* sp. and *Biomphalaria* sp. and their accession numbers selected from the GenBank. **Source:** [27]

**Table 2:** Selected *Bulinus* sp. isolates with their accession numbers on GenBank

S/N	Species	Locality	Country	Accession No	References
1	*B. globosus*	Ngwachani school, Pemba Island	Tanzania	AM 921827	Kane et al., [12]
2	*B. globosus*	Kandaria dam, Kisumu, West Africa (via DBL)^a^	Kenya	AM 286286	
3	*B. globosus*	Pemba Island	Tanzania	AM 921823	Kane et al., [12]
4	*B. globosus*	Kimbuni, Pemba Island	Tanzania	AM 921830	Kane et al., [12]
5	*B. globosus*	Tiengre stream, Kisumu (via DBL)^a^	Kenya	AM 286285	
6	*B. globosus*	Pietermaritzburg (Prof. K.N. De Kock)^a^	South Africa	AM 286289	
7	*B. globosus*	Kinyasini, Unguja Island	Tanzania	AM 286292	
8	*B. globosus*	Lugufu (Dr E. Michel)^a^	Tanzania	AM 286287	
9	*B. globosus*	Road to Mtagani, Pemba Island	Tanzania	AM 921820	Kane et al., [12]
10	*B. globosus*	Ngwachani school, Pemba Island	Tanzania	AM 921826	Kane et al., [12]
11	*B. globosus*	Tiengre stream, Kisumu (via DBL)^a^	Kenya	AM 286284	
12	*B. globosus*	Road to Mtagani, Pemba Island	Tanzania	AM 921825	Kane et al., [12]
13	*B. globosus*	Moyo	Uganda	AM 286291	
14	*B. globosus*	Pietermaritzburg (Prof. K.N. De Kock)^a^	South Africa	AM 286290	
15	*B. globosus*	Kinango	Kenya	AM 921844	
16	*B. globosus*	Kinyasini, Unguja Island	Tanzania	AM 921840	
17	*B. globosus*	Chan-jamjawiri, Pemba Island	Tanzania	AM 921828	Kane et al., [12]
18	*B. globosus*	Thiekeene Hulle	Senegal	AM 921808	
19	*B. africanus*	Isipingo, Durban (Prof. C. Appleton)^a^	South Africa	AM 286296	
20	*B. globosus*	Machengwe, Pemba Island	Tanzania	AM 921829	Kane et al., [12]
21	*B. globosus*	Ipogoro, Iringa	Tanzania	AM 286288	
22	*B. globosus*	Mwaduli	Kenya	AM 921850	
23	*B. globosus*	IRDC farm, Iringa (Dr. S. Walker)^a^	Tanzania	AM 921821	
24	*B. globosus*	Mogtedo barrage	Burkina Faso	AM 286293	
25	*B. globosus*	Kinyasini, Unguja Island	Tanzania	AM 921839	
26	*B. globosus*	Moyo	Uganda	AM 921843	
27	*B. globosus*	Kinango	Kenya	AM 921845	
28	*B. globosus*	Tondia	Niger	AM 286294	
29	*B. globosus*	Kachetu	Kenya	AM 921847	
30	*B. globosus*	Moyo	Uganda	AM 921851	
31	*B. globosus*	Imashayi, Ogun State	Nigeria	KJ361814	Akinwale et al., [23]
32	*B. globosus*	Owode, Ogun State	Nigeria	KF989347	Akinwale et al., [23]
33	*B. truncatus*	Mondego River, Coimbra (Prof. M. Gracio)^a^	Portugal	AM 286314	
34	*B. truncatus*	Mogtedo barrage	Burkina Faso	AM 286315	
35	*B. truncatus*	Mbane	Senegal	AM 921806	
36	*B. truncatus*	Satoni	Niger	AM 286317	
37	*B. truncatus*	Nyanguge	Tanzania	AM 286313	
38	*B. truncatus*	Bouton Batt	Senegal	AM 921807	
39	*B. truncatus*	Posada, Sardinia (Prof. Marco Curini Galletti and Dr. D.T.J Littlewood)^a^	Italy	AM 286312	
40	*B. truncatus*	Satoni	Niger	AM 286316	
41	*B. camerunensis*	Lake Barombi, Kotto	Cameroon	AM 286309	
42	*B. camerunensis*	Owode, Ogun State	Nigeria	KF989354	Akinwale et al., [23]
43	*B. camerunensis*	Ayetoro	Nigeria	KF989356	Akinwale et al., [23]
44	*B, senegalensis*	Ayetoro	Nigeria	KJ361803	Akinwale et al., [23]
45	*B. forskalii*	Ijale Ketu, Ogun State	Nigeria	KF989358	Akinwale et al., [23]
46	*B. forskalii*	Owode, Ogun State	Nigeria	KF989359	Akinwale et al., [23]
47	*B. forskalii*	Katosho swamp, Lake Tanganyika, Tanzania	Tanzania	HQ 121587	Nalugwa, et al., [85]
48	*B. forskalii*	Lake Edward	Uganda	HQ 121583	Nalugwa, et al., [85]
49	*B. forskalii*	Mogtedo barrage	Burkina Faso	AM 286310	Kane, et al., [12]
50	*B. forskalii*	Sao Tome Island, Sao Tome City	Sao Tome & Principe	AM 286305	Kane, et al., [12]
51	*B. forskalii*	Satoni	Niger	AM 286308	Kane, et al., [12]
52	*B. forskalii*	Quifangondo	Angola	AM 286306	Kane, et al., [12]
53	*B. forskalii*	Lake George	Uganda	HQ 121586	Nalugwa, et al., [85]
54	*B. cernicus*	Mont Oreb	Mauritius	AM 286303	
55	*B. barthi*	Kanga swamp, Mafia Island	Tanzania	AM 921817	
56	*B. tropicus*	Njombe Kibena (Dr. S. Walker)^a^	Tanzania	AM 921842	
57	*B. nyassanus*	Kasankha, Money Bay	Lake Malawi	AM 921838	
58	*B. nasutus productus*	Nimbodze	Kenya	AM 921841	
69	*Bulinus* sp.	ADC farm, Kisumu (via DBL)^a^	Kenya	AM 286297	

^a^Contributors Source: www.ncbi/BLAST/index/html

**Table 3:** Selected *Biomphalaria* sp. isolates with their accession numbers on genbank

S/N	Species	Locality	Country	Accession No	References
1.	*Biomphalaria pfeifferi*	Lake Albert	Uganda	EU 141219	Plam et al., [86]
2.	*Biomphalaria pfeifferi*	Ngamilajojo	Uganda	DQ 084834	Plam et al., [86]
3.	*Biomphalaria sudanica*	Ntoroko	Uganda	DQ 084843	Jorgensen et al., [87]
4.	*Biomphalaria pfeifferi*	Kibwezi	Kenya	DQ 084830	Jorgensen et al., [87]
5.	*Biomphalaria glabrata*	Imbaba	Egypt	DQ 084823	Jorgensen et al., [87]
6.	*Biomphalaria pfeifferi*	Lake De Guirs	Senegal	DQ 084831	Jorgensen et al., [87]
7.	*Biomphalaria pfeifferi*	Chiweshe	Zimbabwe	DQ 084829	Jorgensen et al., [87]
8.	*Biomphalaria pfeifferi*	Lwampanga, Lake Kyoga	Uganda	DQ 084833	Jorgensen et al., [87]
9.	*Biomphalaria alexandrina*	Egypt	Egypt	DQ 084825	Jorgensen et al., [87]
10.	*Biomphalaria pfeifferi*	Lwampanga, Lake Kyoga	Uganda	DQ 084833	Jorgensen et al., [87]
11.	*Biomphalaria pfeifferi*	Mansidi port, Lake Kyoga	Uganda	DQ 084841	Jorgensen et al., [87]
12.	*Biomphalaria pfeifferi*	Muzizi	Uganda	DQ 084842	Jorgensen et al., [87]
13.	*Biomphalaria stanleyi*	Lake Albert	Uganda	EU 141215	Plam et al., [86]
14.	*Biomphalaria sudanica*	Lake Albert	Uganda	EU 141227	Plam et al., [86]
15.	*Biomphalaria stanleyi*	Lake Albert	Uganda	EU 141225	Plam et al., [86]
16.	*Biomphalaria sudanica*	Butiaba, Lake Albert	Uganda	DQ 084838	Jorgensen et al., [87]
17.	*Biomphalaria stanleyi*	Butiaba, Lake Albert	Uganda	DQ 084837	Jorgensen et al., [87]
18.	*Biomphalaria sudanica*	Mahyoro	Uganda	DQ 084840	Jorgensen et al., [87]
19.	*Biomphalaria sudanica*	Rutoto	Uganda	DQ 084844	Jorgensen et al., [87]
20.	*Biomphalaria sudanica*	Ntoroko	Uganda	DQ 084843	Jorgensen et al., [87]
21.	*Biomphalaria smithi*	Kwensliama, Lake Edward	Uganda	DQ 084836	Jorgensen et al., [87]
22.	*Biomphalaria camerunensis*	Lake Bakassi	Cameroon	DQ 084827	Jorgensen et al., [87]
23.	*Biomphalaria choanomphala*	Lake Victoria	Uganda	EU 141235	Plam et al., [86]
24.	*Biomphalaria angulosa*	Ruaha River	Tanzania	DQ 084826	Jorgensen et al., [87]
25.	*Biomphalaria smithi*	Rwenshama, Lake Edward	Uganda	DQ 084836	Jorgensen et al., [87]
26.	*Biomphalaria sudanica*	Lake Albert	Uganda	EU 141230	Plam et al., [86]
27.	*Biomphalaria choanomphala*	Lake Albert	Uganda	EU 141226	Plam et al., [86]

Source: www.ncbi/BLAST/index/html

### Snail molecular studies: identification of snail taxa

The use of molecular tools in species identification and exploring host-parasite compatibilities has provided answers to complex evolutionary questions over the years. Though, before the advent of molecular methods in differentiating snail hosts, intermediate snail hosts identification were largely done using morphological descriptions such as shell shape, shell size, nature of aperture, observations on the radula and reproductive system to assess taxonomic variations [28, 29]. However, its applications have enhanced the establishment of database platforms to deepen our understanding on snail hosts diversity and population structure [5, 30]. More importantly, its’ usage in differentiating the complex *Bulinus* group [31] which is the major snail intermediate hosts of *S. haematobium*, a prominent schistosome parasites causing serious morbidity across Africa especially in sub-Saharan Africa.

Advances in the production of effective genetic markers such as random amplified polymorphic DNA (RAPDs) ribosomal gene (rRNA), and the mitochondrial cytochrome oxidase I (COI) have created robust and reliable taxonomy [31] which has improve our knowledge on the epidemiology of schistosomiasis [12].

Though the use of molecular approaches in differentiating snail hosts population structure have been applied on local scales across Africa but it is yet to be fully explored. For instance, [14] identified *B. forskalii*, *Bi. pfeifferi* and *B. truncatus* using molecular methods in N’Djamena, Chad [22]. Comprehensively identified five snail hosts (*B. globosus, B. forskalii, Bi. pfeifferi, Lymnaea natalensis* and *Indoplanorbis exutus*) of trematode parasites in Nasarawa State, north central, Nigeria using mitochondrial gene cytochrome oxidase I (*cox1*). The study assessed the phylogenetic relationship of these snails and established that *B. globosus* from Nasarawa State, Nigeria clusters with *B. globosus* sequence data from other West African countries such as Burkina Faso, Senegal and Niger when BLAST, using nucleotide blast homology on genbank forming a monophyletic lineage but forms paraphyletic relationship with *B. globosus* species from East Africa. *B. forskalii* also followed similar pattern, as it cluster to form a monophyletic relationship with species from Burkina Faso (Mogtedo barrage), Niger (Tondia) and Senegal (Thiekeene Hulle) while *Bi. pfeifferi* from Nigeria clustered with *Bi. pfeifferi* species from Senegal (Lake De Guirs), Kenya (Kibwezi), Uganda (Lake Albert) and Zimbabwe (Chiweshe) to form a monophyletic relationship. *Indoplanorbis exutus* formed a paraphyletic relationship with species from Asia. Information is lacking on the phylogenic status of *Indoplanorbis exutus* and *Lymnaea natalensis* from Africa, there is need to prioritize the establishment of reliable genome database for these snails across Africa considering their veterinary importance. Similarly, [32] characterized *Bulinus truncatus* using PCR-RFLP technique and assessed their infection status with *Dra I* gene repeat in Southwest Nigeria while [23] established the population structure of *B. globosus, B. forskalii, B. camerunensis* and *B. senegalensis* in schistosomiasis endemic communities of Ogun state, Southwestern Nigeria through the application of PCR-RFLP on the snails ribosomal ITS region.

Molecular tools application is not limited to elucidating relationships across snail hosts taxa. The application of PCR *DraI* and sm17 in the early detection of *S. haematobium* and *S. mansoni* in infected snail intermediate hosts of the *Bulinus sp.* and *Bi. pfeifferi* respectively have helped strengthen snail surveillance and boost schistosomiasis control efforts [33, 34]. Also, the simultaneous usage of PCR, *DraI* PCR and Sh110 SmSl PCR were effective in differentiating schistosome parasites that infected snails within the *Bulinus* group in Morocco [35]. Table 4 shows the summary of intermediate snail host studies carried out in different parts of Africa.

**Table 4:** Summary of snail intermediate hosts studies in different parts of Africa

S/N	Country	Snail species	References
1.	Nigeria	*Bulinus globosus*	[13, 22]
		*Bulinus forskalii*	
		*Biomphalaria pfeifferi*	
		*Lymnaea natalensis*	[16]
		*Indoplanorbis exutus*	
		*Bulinus* sp.	[23]
2.	Chad	*Bulinus truncatus*	[14]
		*Bulinus forskalii*	
		*Biomphalaria pfeifferi*	
3.	Angola	*B. globosus*	[77]
		*B. canescens*	
		*B. angolensis*	
		*B. crystallinus*	
		*Bi, salinarium*	
		*B. globosus*	
		*B. canescens*	
4.	Egypt	*Biomphalaria alexandrina*	[37]
			[38]
		*Lymnaea natalensis*	[88]
		*Bulinus truncatus*	
		*Bulinus truncatus*	[39]
5.	Cameroon	*Bulinus truncatus*	[39]
		*B. globosus*	
		*B. senegalensis*	
		*B. tropicus*	
		*B. forskalii*	
		*B. camerunensis*	
		*B. globosus*	[15]
		*B. forskalii*	[11]
6.	Senegal	*Bulinus truncatus*	[39]
		*B. senegalensis*	
		*B. umbilicatus*	
7.	Lake Victoria (across Tanzania, Kenya and Uganda)	*Biomphalaria choanomphala*	[40]
8.	Tanzania	*B. globosus*	[89]
9.	Madagascar	*Biomphalaria pfeifferi*	[44]
10.	Malawi	*B. globosus*	[90]
		*B. nyassanus*	
11.	Cote D’ voire	*B. forskalii*	[11]
		*B. globosus*	
12.	Equitorial Guinea	*B. forskalii*	[11]
13.	Niger	*B. umbilicatus*	[11]

### Snail genome studies: implication for effective control programme

The need to meet the goals of schistosomiasis elimination prompted the pursuit of an integrated control approach [3, 36] and contributions from different stakeholders [31] have provided baseline information and vigor for the pursuit of efficient implementation of control efforts in Africa [15, 37–40].

It is observed that environmental factors play significant role in the population size of snail host’s natural populations. The effects of these environmental conditions greatly affect gene flow between populations and induces important variations in population size [29]. Their hermaphroditic capabilities enable self or cross fertilization and allows for different genetic consequences [41]. Also, the fitness impact of parasites on the snail mating systems affects the genetic structure of the snail hosts population [42, 43]. Good understanding of local fluctuation in geographic origin, population size and snail hosts’ reproductive potentials are fundamental to improving our knowledge on the demographic stochasticity of natural population’s genetic structure [43].

The investigation of snail genetics role in trematode parasite infections variation using molecular approaches is vital to understanding their epidemiology. The assessment of the genetic differentiation of *Bulinus* snails from different ecological zones across Cameroon, Egypt and Senegal revealed high genetic diversity within *Bulinus* populations sampled from the three countries with the highest diversity observed within populations of *B. forskalii* and *B. senegalensis* [39], but this is contrary to findings on *Biomphalaria pfeifferi* in Madagascar which was reported to have high level of inter-population variation [44]. Utilizing the use of molecular markers[45], showed that there was high intra-population diversity with high levels of population structure but low level gene flow among populations of *Biomphalaria choanomphala* along Lake Victoria covering Tanzania, Kenya and Uganda. The study identified consistent parasitism as the major influencing factor [46] indicated that *Biomphalaria* species of African origin were derived from the neotropical natives and that proto-*Biomphalaria glabrata* is the progenitor of the African species through the trans-Atlantic colonization of Africa.

Findings have shown that schistosome parasites development inside the snail host is influenced by both the host and parasite genes [17, 47]. This has increase stakeholders consciousness to unsnarl the schistosome parasites and snail genes that influence this intrinsic association [48, 49]. This led to the development of genetic markers for the identification of resistant genes within the snail hosts. Detailed elucidation of snail hosts population structure and the identification of genetic markers for parasite resistance will further boost the resolve of effective integrated control approach for schistosomiasis elimination in Africa [37]. Observed from investigation on identified refractory strains to *S. mansoni* in *Bi. alexandrina* population from Egypt that refractory character within the snail hosts population is hereditary and therefore advised that snails that are actively resistant to schistosome parasites should be cultured to encourage biological control of snail intermediate hosts in a natural population.

Furthermore, it was established that snail hosts infection with schistosome parasites is species specific and often localized [50], efforts should be made to identify and document snail hosts that have refractory characters across regions. The introduction of snail hosts with parasite resistant genes into the natural population to replace the susceptible snail species in endemic areas will discourage schistosomiasis transmission and also reduce damage to the natural ecosystem through the use of molluscicides.

More importantly, it is necessary to encourage the extensive study of snail genome differentiation on a large scale due to the current global changes that have led to changes in the modification of the geographical distribution of species prompting hybridization, such hybridization is already known to occur in schistosomes and offspring have been shown to have superior virulence and invasive capacities [51]. This is an emerging public health concern particularly because of the changing geographic distribution of humans, domestic animals and wildlife [52]. Prioritizing snail studies is essential and there is need to update the snail hosts genome library for Africa in order to boost the realization of schistosomiasis elimination through active integrated control mechanisms. This is important because of the dynamic changes in climatic and environmental conditions which play key roles in the distribution of the snail intermediate hosts and the development of schistosome parasites.

Large-scale assessment of snail intermediate hosts genome will create the platform to determine the degree of variability among and within snail populations across the continent and give an overview of schistosomiasis distribution in Africa with current realities.

**Fig. 3:**
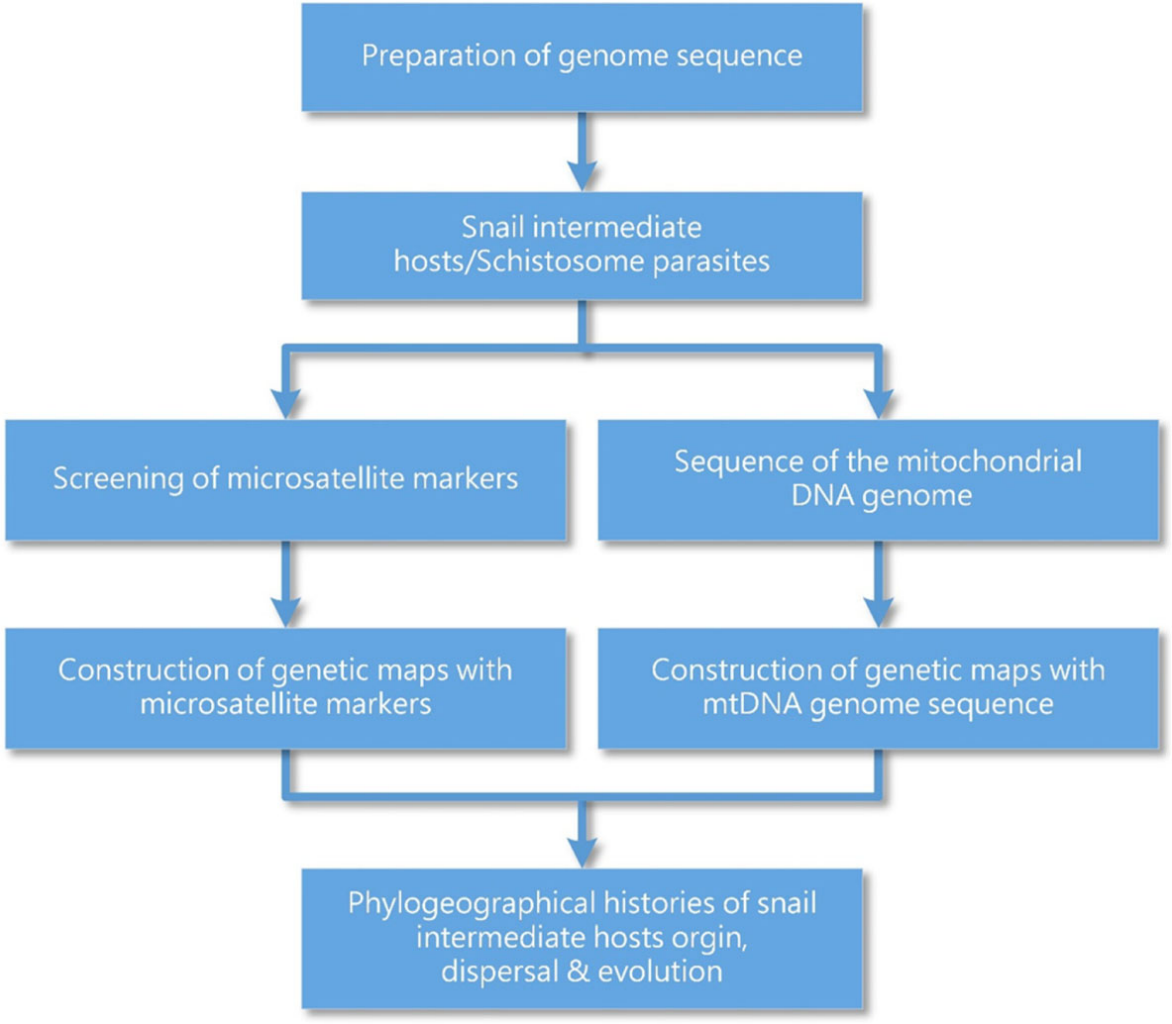
Determination of snail intermediate hosts population genetics and diversity using biomarkers

Determination of snail intermediate hosts population genetics and diversity using biomarkers is shown in Fig. 3

### Gaps analysis: three research priorities identified

Though, molecular approaches to differentiating snail intermediate hosts are key to combating the menace of this debilitating disease in Africa [17]. However, studies on snail biology should not be limited to the application of molecular methods because there are other aspects of snail studies that are essential and should be taken seriously.

Firstly, snail identification using shell morphology and bionomics studies are essential to understanding the distribution pattern of snail hosts and transmission dynamics of schistosomiasis and other disease causing snail-borne trematodes at local scales across the continent [53, 54]. We observed that there is dearth of knowledge and lack of expertise in the area of malacology in Africa, this might be due to lack of interest from individuals as it is believed that the application of molecular methods is more acceptable and efforts are geared towards establishing collaborations that will help access such platforms. However, the knowledge and expertise of snail identification using shell morphology requires highly trained professionals to enhance capacity building due to its importance in disease surveillance and should be prioritized in order to achieve the goal of schistosomiasis elimination in Africa.

Secondly, the application of Geographical Information System (GIS) and remote sensing technologies to map and define the spatial limits of snail hosts distribution is an important area that requires utmost attention. Though it has been applied in some parts of Africa on local scales [55–57], but information on the geospatial distribution of important snail intermediate hosts is lacking in most African countries. Mapping and predicting snail hosts distribution on national and continental scales to establish comprehensive GIS database will help characterize the different eco-zones with relevance to the prevalent diseases, thus provide information that will enhance optimizing the use of available resources [58], and also strengthen the drive for effective schistosomiasis control on the continent.

Thirdly, there is urgent need to aggresively create awareness by educating the larger society especially people in the endemic areas through the mass media and other communication platforms on the importance of these planorbid snail hosts in schistosomiasis epidemiology. Experience in the field have shown that most locals who live around waterbodies in most endemic settings have little or no knowledge of the snails and are not aware of the danger their presence poses to their well-being. Hence, it is important to consistently create awareness on snail hosts control. The locals should be equipped with information that will spur them to ensuring that the snails does not thrive in their environment and also be mandated to urgently report snail hosts presence in any waterbody around their domain to the relevant health authorities promptly.

In addition to the aforementioned three gaps on snail biology, there are other areas that requires attention. This includes effort to put infrastructure in place or consistently modify the environment to discourage the continued presence and distribution of snail hosts and schistosomiasis transmission in most endemic countries. The environment in most endemic countries are characterized by factors that influence the distribution of snail hosts of schistosome parasites as a result of poor environmental management [59–62]. The presence of aquatic plants such as *Eichhornia crassipes* within and around waterbodies enhance the occurrence, distribution and abundance of snail hosts because it serves as a good source of food, provide shelter and oviposition sites for the snails [63–65]. Environmental modification through active removal of aquatic plants and silts from waterbodies renders the habitat unfriendly to the snail hosts [66, 67]. The indiscriminate disposal of human wastes due to lack of sanitary facilities and poor access to potable water sources for domestic purposes also add to the sources of infection in the environment, this facilitates easy access of schistosome parasites in feces or urine from infected persons to waterbodies and snail intermediate hosts.

Despite the public health significance of schistosomiasis globally especially in Africa where about 95% of global schistosomiasis is concentrated [68, 69], the use of micro-array platforms to decipher the intricate interplay between the parasites and the snail hosts is scarce. There is need for drastic improvement in the application of immunomic and next-generation sequencing platforms regarding schistosomiasis and other NTDs [70–75]. Efforts should be geared towards identifying genes that are actively involved in snail’s immune responses in order to initiate defence mechanisms that will block schistosome parasites survival in the snails [76]. Molecular tools application is vital for efficient snail surveillance and has great potential, as it is important for snail hosts and trematode parasites identification and also useful in defining the level of species biodiversity [5, 22, 31, 77]; these are pre-requisite to blocking schistosomiasis transmission effectively [78]. The lack of reference laboratories to carry out early diagnosis of schistosomiasis cases on infected people is a big challenge to the pursuit of schistosomiasis elimination in Africa. This debacle also extends to poor or absence of platform for researchers to execute evidence-based research on snail hosts. Such platforms, if available would help strengthen schistosomiasis surveillance and capacity building within the continent.

The challenge of insufficient supply of praziquantel due to scarcity of funds and the resistance of schistosome parasites to the drug of choice [79] led to the increasing call for the use of molluscicide to curtail snail distribution, but molluscicide application is yet to be substantially utilized in many countries endemic for schistosomiasis in Africa. This is partly due to reliance on prioritized chemotherapy treatment of school-aged children with praziquantel, which is not very effective due to the high re-infection rate few weeks after treatment or due to insensitivity or poor knowledge about snail hosts’ role in schistosomiasis transmission.

This might also be attributed to the perceived negative impact that niclosamide, the molluscicide of choice have on fishes, an important protein source and means of generating income for people living in rural settings. Therefore, it is advised that the molluscicide formulation be improved to ensure that it has less negative impact on the environment and biodiversity [80], but retain its potency against snail hosts [81].

The exploration of the molluscicidal properties of plants such as *Phytolacca dodencandra* and *Millettia thonningii* and some other plants with similar properties [82] should be considered*.* The distribution of these molluscicidal plants in areas identified as schistosomiasis hotspots in endemic areas will help curtail the distribution of snail hosts. However, it is important to effectively monitor the plants when cultivated in large scale because of their toxic properties.

**Fig. 4:**
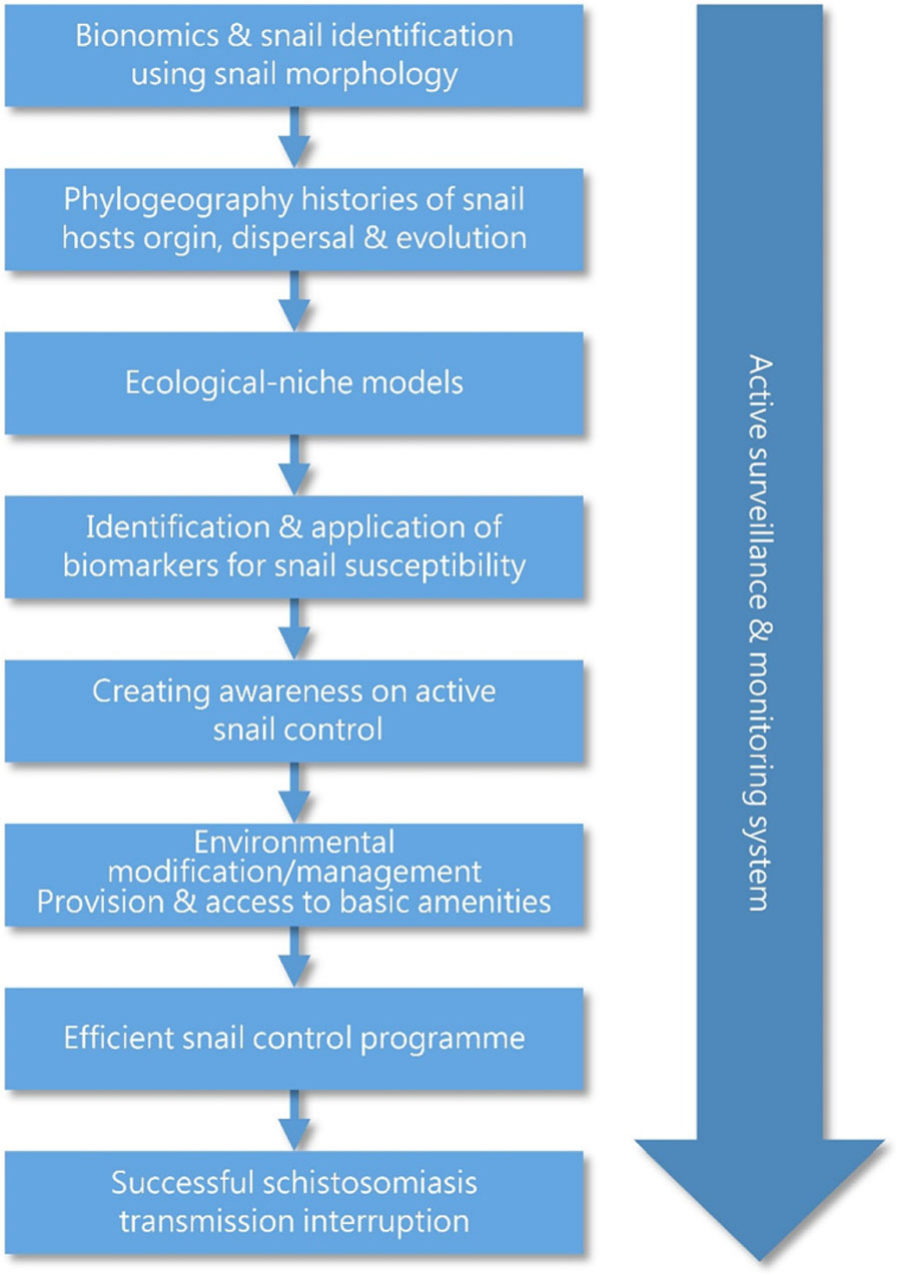
Mechanism for efficient schistosomiasis transmission interruption

The use of biological techniques for snail hosts control is long overdue in Africa, measures should be taken to effectively apply natural predators or encourage biotechnological methods to induce infecundity in the snails [83]. Figure 4 shows the mechanism for efficient schistosomiasis transmission interruption.

## Conclusions

The elimination of schistosomiasis and other trematode parasite infections will receive a great boost when snail hosts studies and effective snail control programme are prioritized. There is urgent need to set-up reference laboratories and other platforms that will encourage qualitative snail intermediate hosts and schistosomiasis researches and also facilitate early diagnosis of schistosomiasis cases. It is imperative to encourage capacity building through training and re-training of scholars, health workers and different stakeholders in Africa on snail hosts identification using both morphological and molecular approaches.

## Additional file

Additional file 1: Multilingual abstracts in the four official working languages of the United Nations. (PDF 514 kb)

## Abbreviations

DNA: Deoxyribonucleic acid
RAPD: Random Amplified Polymorphic DNA
rRNA: ribosomal ribonucleic acid
COI: Cytochrome oxidase I
PCR-RFLP: Polymerase Chain Reaction- Restriction Fragment Length Polymorphism
ITS: Internal Transcribed Spacer
GIS: Geographical Information System
