# Supplementary material for: Differentiating snail intermediate hosts of Schistosoma spp. using molecular approaches: fundamental to successful integrated control mechanism in Africa
Source: Infect Dis Poverty. 2018 Mar 26;7:29. doi: 10.1186/s40249-018-0401-z (PMC5883423; doi:10.1186/s40249-018-0401-z)
Supplement: Supplementary file 1 — Multilingual abstracts in the four official working languages of the United Nations. (PDF 514 kb) [file 40249_2018_401_MOESM1_ESM.pdf]

Translation of the abstract into the five official working languages of the United Nations

تحديد دور الحلزون كمضيف انتقالي لدودة البلهاريسيا عبر استخدام المقاربة الجزيئية: خطوة ضرورية في سبيل السيطرة على مرض البلهاريسيا في أفريقيا

Eniola Michael Abe, Wei Guan, Yun-Hai Guo, Kokouvi Kassegne, Zhi-Qiang Qin, Jing Xu[1], Jun-Hu Chen, Uwem Friday Ekpo, Shi-Zhu Li and Xiao-Nong Zhou

ملخص

الإطار العام: يلعب الحلزون كمضيف انتقالي دورا هاما في انتشار التهابات تريماتود الحلزونية المنقولة في أفريقيا. إن الدراسة المعمقة للأمراض المنقولة بواسطة الحلزون، خاصة بوصفه مضيفا انتقاليا، ستساهم في تعزيز الجهود الرامية لاحتواء تلك الأمراض.

جوهر الموضوع: تسلط هذه الدراسة الضوء على أهمية المقاربة الجزيئية في تحديد دور الحلزون كمضيف انتقالي لدودة البلهاريسيا، وعلى وجوب توفير المعلومات الضرورية حول دور الحلزون كمضيف انتقالي لدودة البلهاريسيا ، وذلك عن طريق تحديث قاعدة بيانات جينوم الحلزون كمضيف انتقالي لدودة البلهاريسيا بالنسبة لقارة أفريقيا. تهدف هذه الجهود إلى تزويد مختلف الأطراف الفاعلة بالمعلومات اللازمة حول البيئة التي يعيش فيها الحلزون كمضيف انتقالي لدودة البلهاريسيا ، ودوره في نقل عديد الأمراض. أيضا نقوم بتحديد الثغرات والمجالات التي تستدعي تدخلا عاجلا من أجل تحقيق السيطرة التامة على مرض البلهاريسيا وباقي التهابات تريماتود الحلزونية المنقولة.

الخلاصة: الاهتمام بتحديد دور الحلزون كمضيف انتقالي لدودة البلهاريسيا ، خاصة عن طريق المقاربة الجزيئية، سيمكن من دعم جهود احتواء مرض البلهاريسيا في أفريقيا.

Translated from English version into Arabic by Maher Abassi, through

TRANSLATORS
WITHOUT BORDERS

Differentiating snail intermediate hosts of *Schistosoma* spp. using molecular approaches: fundamental to successful integrated control mechanism in Africa

Eniola Michael Abe, Wei Guan, Yun-Hai Guo, Kokouvi Kassegne, Zhi-Qiang Qin, Jing Xu[1], Jun-Hu Chen, Uwem Friday Ekpo, Shi-Zhu Li and Xiao-Nong Zhou

摘要

引言：在非洲，螺作为中间宿主在螺源性吸虫病的传播中扮演着积极重要的作用。充分了解螺源性疾病，尤其是螺群体的流行病学特征将推动当前螺源性疾病控制策略的进一步完善。

主要内容:本综述强调了分子方法在鉴别非洲螺种群结构中的重要性，并阐述了通过更新螺基因组数据库提供准确螺种群信息的必要性，从而为各利益群体提供有关螺中间宿主的生态学及其在不同疾病传播中作用的充足信息。同时，我们确定了急需干预以促进血吸虫病及其他螺源性吸虫传染病综合有效控制的区域及目前存在的差距。

结论：优先开展螺相关研究，尤其是采用分子工具开展螺群鉴别研究不仅将推动非洲疾病监测工作，同时可促进血吸虫病防治项目的有效运行。

Дифференциация улиток как промежуточного хозяина *шистосом (Schistosoma* spp.) с помощью молекулярных подходов: основополагающих для успешного комплексного механизма контроля в Африке

Эниола Майкл Абе, Вэй Гуань, Юнь-Хай Гуо, Ковуви Кассень, Чжи-Цян Цинь, Цзин Сюй[1], Цзюнь-Ху Чэнь, Увем Фрайдей Экпо, Ши-Чжу Ли и Сяо-Нон Чжоу

Аннотация

Справочная информация: Улитка выполняет активную роль промежуточного хозяина при передаче трематодных инфекций, переносимых улитками в Африке. Хорошее знание эпидемиологии переносимых улитками заболеваний, особенно в случаях, когда улитки выполняют роль промежуточного хозяина популяций, способно обеспечить необходимый импульс, дополняя уже существующую стратегию контроля.

Основная часть: В настоящем обзоре подчёркивается важность молекулярных подходов при дифференциации структур популяции улиток в качестве переносчиков инфекции, а также необходимость предоставления надлежащей информации по популяции улиток-переносчиков инфекции путём обновления геномной базы данных популяции улиток по Африке с целью предоставления различным заинтересованным лицам и группам надлежащей информации по экологии улиток в качестве промежуточного хозяина, а также их по их роли в передаче различных заболеваний. Аналогично, выявлены пробелы и области, по которым требуется незамедлительное вмешательство с целью содействия эффективному и всеобъемлющему контролю за шистосомозом, а также за другими трематодными инфекциями, переносимыми улитками.

Выводы: Уделение приоритетного внимание изучению улиток, в особенности дифференциации улиток с применением молекулярного инструментария, улучшит наблюдение за заболеваемостью, а также повысит эффективность выполнения программы по контролю за шистосомозом в Африке.

Translated from English version into Arabic by Liudmila Tomanek, through

TRANSLATORS
WITHOUT BORDERS

La différenciation des escargots servant d'hôtes intermédiaires à *Schistosoma* spp. par les méthodes moléculaires : une méthode fondamentale pour réussir les mécanismes de lutte intégrée en Afrique.

Eniola Michael Abe, Wei Guan, Yun-Hai Guo, Kokouvi Kassegne, Zhi-Qiang Qin, Jing Xu[1], Jun-Hu Chen, Uwem Friday Ekpo, Shi-Zhu Li and Xiao-Nong Zhou

Résumé

Contexte : Les escargots servant d'hôtes intermédiaires aux trématodes participent activement à la

transmission des infestations véhiculées par les gastéropodes en Afrique. Une bonne connaissance de l'épidémiologie de ces maladies, et notamment des populations des hôtes intermédiaires, pourrait donner une nécessaire impulsion supplémentaire à la stratégie de lutte existante.
Discussion : Cette revue souligne l'importance des méthodes moléculaires dans la différenciation de la structure des populations de gastéropodes hôtes et la nécessité de fournir des informations adéquates sur ces populations en actualisant la base de données de leur génome en Afrique, de façon à fournir aux différentes parties prenantes des informations adéquates sur l'écologie de ces escargots et leur rôle dans la transmission de diverses maladies. Nous identifions également les lacunes et les domaines où une intervention est nécessaire d'urgence pour une lutte intégrée efficace contre la schistosomiase et autres infestations à trématodes véhiculées par les escargots.
Conclusions : La priorité donnée aux études sur les escargots, et notamment à la différenciation de ceux-ci au moyen d'outils moléculaires, dynamiserait la surveillance des maladies et renforcerait un programme efficace de lutte contre la schistosomiase en Afrique.

Translated from English version into French by Suzanne Assenat, through

TRANSLATORS
WITHOUT BORDERS

Diferenciar el caracol huésped intermedio del *Schistosoma* spp usando enfoques moleculares, fundamental para un mecanismo de control integrado exitoso en África

Eniola Michael Abe, Wei Guan, Yun-Hai Guo, Kokouvi Kassegne, Zhi-Qiang Qin, Jing Xu[1], Jun-Hu Chen, Uwem Friday Ekpo, Shi-Zhu Li and Xiao-Nong Zhou

Resumen

Antecedentes: los caracoles huéspedes intermedios desempeñan un papel activo en la transmisión de las infecciones por trematodos transmitidas por caracoles en África. Un conocimiento adecuado de la epidemiología de las enfermedades transmitidas por caracoles, en particular las poblaciones de huéspedes intermedios, brindaría el estímulo necesario para complementar la estrategia de control existente.
Cuerpo principal: Esta revisión resalta la importancia de los enfoques moleculares para diferenciar la estructura de la población de caracoles huéspedes y la necesidad de proveer información adecuada sobre las poblaciones de caracoles huéspedes intermedios actualizando la base de datos para África del genoma de los caracoles huéspedes. De este modo se equiparía a las distintas partes interesadas con información adecuada sobre la ecología de los caracoles huéspedes intermedios y su rol en la transmisión de distintas enfermedades. Asimismo, identificamos las brechas y las áreas donde se necesita una intervención urgente para facilitar el control integrado y efectivo de la esquistosomiasis y otras infecciones transmitidas por caracoles.
Conclusiones: Priorizar los estudios de caracoles, especialmente la diferenciación de caracoles usando herramientas moleculares promoverá la vigilancia de enfermedades y también reforzará el programa de control eficiente de la esquistosomiasis en África.

Translated from English version into Spanish by Lidia Lariño, through

TRANSLATORS
WITHOUT BORDERS
